# Agile marketing in ophthalmological organizations

**DOI:** 10.22336/rjo.2022.40

**Published:** 2022

**Authors:** Consuela-Mădălina Gheorghe

**Affiliations:** * Philologist, Authorized translator

The agile concept represents a set of management principles that developed originally from the software industry and offers the healthcare leaders an opportunity to approach most of the challenges in the healthcare management. Agile marketing appeared because of the necessity to consolidate the processes of the organizations in software development. In addition, today, agile helps leaders in many fields such as marketing, finance and healthcare. 

Agile marketing is a form of marketing that makes use of agile methodologies' guiding ideas and procedures. Self-organizing, cross-functional teams that operate in regular iterations with ongoing input are examples of this. It necessitates short-, medium-, and long-term marketing planning in addition to a strategic vision (https://www.atlassian.com/agile/agile-marketing/what-is-agile-marketing).

In the context of marketing, agile refers to the constant use of information and analytics to determine interesting prospects or solutions to issues in real time, the rapid deployment of tests, the evaluation of the findings, and the rapid emphasis of results. An agile marketing department in an ophthalmological organization that acts at scale can manage numerous new concepts each week, as well as hundreds of campaigns that are running concurrently (https://www.mirabelle.co.uk/agile-marketing/).

Agile marketing is a form of advertising that borrows from agile software development and advocates for the following principles: adapting to change versus sticking to a plan; swift revisions as opposed to great campaigns; test results and statistics over beliefs; several little tests instead of a few big bets; individuals and interactions rather than a universal model cooperation instead of ranking and archives (https://www.atlassian.com/agile/agile-marketing/what-is-agile-marketing).

The objectives of agile marketing are to increase the marketing function's speed, predictability, transparency, and receptivity to change.

Agile marketing was created in response to the evolution of marketing strategies over the past 30 years. To reach a wide audience, marketing teams have traditionally employed media including radio, print advertising, and billboards. Once or twice a year, they made significant time and financial investments to launch a significant campaign. Additionally, the instruments they had to gauge return on investment were scarce (https://www.aha.io/roadmapping/guide/marketing-methods/what-is-agile-marketing).

However, the way marketing teams in ophthalmological organizations work has changed thanks to the internet. Customers in the ophthalmological field are now primarily reached through digital marketing channels including social media, search engines, email, and online display advertising. Through these channels, marketing teams can get a lot of information about customer behavior and learn insightful things about their preferences (https://www.investopedia.com/terms/d/digital-marketing.asp).

Therefore, to keep up with our rapidly evolving digital world, the marketing teams in the ophthalmological organizations must work in a more gradual, measurable, and adaptable manner. Because of this, lots of ophthalmological organizations are starting to use agile marketing. Teams can be more agile, provide more individualized messages, maintain competition, and provide better outcomes for the ophthalmological organization (https://www.atlassian.com/agile/agile-marketing/what-is-agile-marketing).

Adopting an agile marketing strategy in an ophthalmological organization has other advantages, such as integrated campaigns and programs, saving money, data-driven judgements and trust and openness (https://www.mckinsey.com/capabilities/growth-marketing-and-sales/our-insights/agile-marketing-a-step-by-step-guide). 

In addition, the partners in digital, content, and product marketing agree with the marketing strategies and can provide new, present and future consumers with a unified, smooth experience.

Moreover, by emphasizing, the ophthalmological organizations avoid wasting time and misusing resources on strategies that are improper for their target audience. For instance, they can promptly stop investing in a channel if it is not producing the desired outcomes.

Monitoring real-time data empowers the ophthalmological organization to gain smart knowledge of their audience attitude. Traffic, clicks, and conversion rates produce results about the validity of the ophthalmological organization programs and campaigns and disclose what must be changed to productively reach its target audience.

The collaboration and constant communication within the ophthalmological marketing teams ensure that team members' abilities are equitably distributed among them and that everyone's skills are used to their full potential.

Agile marketing differs from conventional marketing because it targets regular releases, it is based on intentional experimentation and it mainly focuses on the contentment of the ophthalmological consumers. 

In conclusion, the benefits of agile marketing in an ophthalmological organization associate the success and effectiveness of the ophthalmological team in replying the patients’ demands. At the same time, agile process allows partners to concentrate more on responsibilities rather than on roles. Agile offers the ophthalmological teams the opportunity to innovate by authorizing teams to assess and address problems.

The phacoemulsification system is based on cataractous lens emulsification with ultrasonic energy transformed by electric energy. The Centurion system (Alcon, Fort Worth, TX) gives an opportunity to monitor ultrasonic energy delivered into the eye as cumulative dissipated energy (CDE). Cataract patients with corneal opacity are considered challenging cases, and some auxiliary methods or surgical manipulations and ultrasonic energy out of standard can be required during surgery. The purpose of this study was to compare ultrasonic energy delivered into the eye, CDE values, and frequencies of required auxiliary surgical methods during phacoemulsification and IOL implantation procedure, between eyes with and without corneal opacity.

